# Validity of a Fully-Immersive VR-Based Version of the Box and Blocks Test for Upper Limb Function Assessment in Parkinson’s Disease †

**DOI:** 10.3390/s20102773

**Published:** 2020-05-13

**Authors:** Edwin Daniel Oña, Alberto Jardón, Alicia Cuesta-Gómez, Patricia Sánchez-Herrera-Baeza, Roberto Cano-de-la-Cuerda, Carlos Balaguer

**Affiliations:** 1 Department of Systems Engineering and Automation, University Carlos III of Madrid, Avda. de la Universidad 30, 28911 Leganés, Spain; ajardon@ing.uc3m.es (A.J.); balaguer@ing.uc3m.es (C.B.); 2 Department of Physical Therapy, Occupational Therapy, Rehabilitation and Physical Medicine, Rey Juan Carlos University, Avda. de Atenas s/n, 28922 Alcorcón, Spain; alicia.cuesta@urjc.es (A.C.-G.); patricia.sanchezherrera@urjc.es (P.S.-H.-B.)

**Keywords:** automatic, assessment, manual dexterity, Parkinson’s disease, neurological rehabilitation, virtual reality, games for health

## Abstract

In recent decades, gaming technology has been accepted as a feasible method for complementing traditional clinical practice, especially in neurorehabilitation; however, the viability of using 3D Virtual Reality (VR) for the assessment of upper limb motor function has not been fully explored. For that purpose, we developed a VR-based version of the Box and Blocks Test (BBT), a clinical test for the assessment of manual dexterity, as an automated alternative to the classical procedure. Our VR-based BBT (VR-BBT) integrates the traditional BBT mechanics into gameplay using the Leap Motion Controller (LMC) to capture the user’s hand motion and the Oculus Rift headset to provide a fully immersive experience. This paper focuses on evaluating the validity of our VR-BBT to reliably measure the manual dexterity in a sample of patients with Parkinson’s Disease (PD). For this study, a group of twenty individuals in a mild to moderate stage of PD were recruited. Participants were asked to perform the physical BBT (once) and our proposed VR-BBT (twice) system, separately. Correlation analysis of collected data was carried out. Statistical analysis proved that the performance data collected by the VR-BBT significantly correlated with the conventional assessment of the BBT. The VR-BBT scores have shown a significant association with PD severity measured by the Hoehn and Yahr scale. This fact suggests that the VR-BBT could be used as a reliable indicator for health improvements in patients with PD. Finally, the VR-BBT system presented high usability and acceptability rated by clinicians and patients.

## 1. Introduction

Parkinson’s disease (PD) is a progressive nervous system disorder with motor and non-motor signs and symptoms [1,2]. Among motor disorders, PD is characterized by bradykinesia, rigidity, resting tremor, and postural instability. In addition, patients with PD typically suffer from a wide range of non-motor problems as orthostatic hypotension, sleep disorders, pain, fatigue, excessive sweating, urinary disorders, loss of sense of smell, sexual problems, or mood and cognitive changes [3]. At present, there is no curative treatment for PD, so pharmacological and rehabilitation treatments are standard to manage this neurodegenerative disorder [4,5]. Even from early stages of the disease, dexterity is frequently disordered and one of the contributors to the burden of the disease [6]. Dexterity, coordination, and gross and fine motor control in patients with PD impair basic activities of daily living (ADL) [6]; as such, patients become more dependent on their caregivers.

In this context, evaluating the progress of the disease or the effectiveness of an intervention are particularly important in PD [7]. Several standardized tools can be used to measure different functional aspects of extremities [8]. Among upper extremity dexterity assessment, many tools have been described [9,10], such as the Jebsen Taylor Test of Hand Function, Nine Hole Peg Test, Purdue Pegboard, Crawford Small Parts Dexterity Test, Functional Dexterity Test, Grooved Pegboard, Moberg Pick-Up Test, Minnesota Rate of Manipulation Test, O’Connor Finger Dexterity Test, and the Sequential Occupational Therapy Dexterity Assessment. One of the most commonly used tests for upper extremity (UE) evaluation is the Box and Blocks Test (BBT) [11,12]. The BBT measures unilateral gross manual dexterity, and it can be used for different populations, including elderly subjects with upper limb disability [12]. The BBT is a wooden box divided into two compartments and 150 blocks. The test consists of moving, one-by-one, the maximum number of blocks from one compartment of the box to another of equal size, within 60 s. The test begins with the unaffected UE to register scores, and both sides are tested. The BBT has shown its validity in subjects with UE disability.

Currently, virtual reality (VR) has become extremely relevant in the rehabilitation of cognitive and motor disorders in patients with PD [13,14]. VR offers a highly motivating multidimensional virtual environment which the user can interact with, which provides sensorial feedback, thus allowing the individual to function in activities or tasks comparable to real situations. The degree of intensity and difficulty can be graded and provides the user with information about the objectives reached in real-time [15,16]. Therefore, it is a new rehabilitation tool with a wide range of potential applications, including functional assessment [17,18,19,20,21]. In this way, the opportunities of using VR for the assessment of UE motor function have not been widely exploited. Some studies have developed virtual versions of the BBT using the Kinect [22] or Leap Motion Controller (LMC) [23]. These systems have been slightly tested with stroke survivors and healthy students, respectively, having good results; however, common limitations of such systems are the reduced depth perception (a flat-screen monitor) and the lack of automatic test administration. Consequently, there is still room for improvements.

On account of the above, in our previous work [24], we implemented a virtual system for automatic assessment of manual dexterity based on the BBT, denoted as the VR-based Box and Blocks Test (VR-BBT). This system was developed based on the assumption that combining a reliable hand motion tracking with a fully-immersive VR setting can lead to better user’s performance. Thus, the VR-BBT uses the LMC for robust tracking of hand gestures and the Oculus Rift headset to provide the user with better depth perception in a fully-immersive environment. This system was preliminarily tested for the first time with a sample of patients with PD [25], obtaining promising results that keep some similarities to studies conducted in other populations.

The objective of this research was to study the validity, feasibility, and psychometric properties of a fully immersive VR-BBT to assess manual dexterity in patients with PD. For that goal, a sample of twenty individuals with PD were recruited. In the trial, participants performed the physical BBT first (once), and then our VR-BBT system (twice). The correlation level between the scores from the physical BBT and virtual systems was calculated in order to quantify the reliability of outcomes. Besides, the test–retest reliability was studied for the two attempts with the VR-BBT.

The remainder of this article is organized as follows: Section 2 describes the BBT characteristics (components and method) and the related work in this field. Section 3 presents the proposed VR-BBT and describes the main features of the system. Section 4 describes the pilot trial conducted to evaluate the performance of the VR-BBT system with patients with PD. The methods used for usability and statistical analysis are also presented. Section 5 includes the results of the pilot trial. Additionally, the acceptance of the proposed tool is studied using therapists’ and patients’ satisfaction questionnaires. Section 6 discusses the performance and feasibility of the proposed system as an alternative to traditional outcome measures. Finally, the conclusions are summarized in Section 7.

## 2. Background

**Figure 1:**
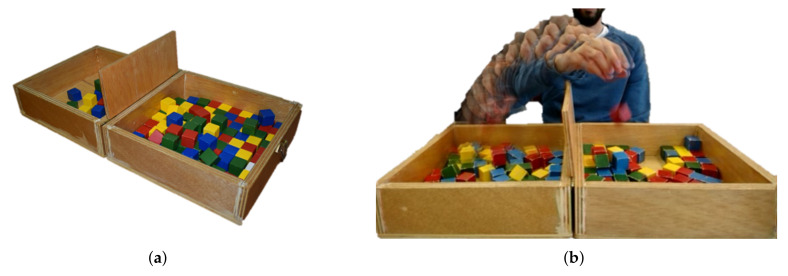
The classical Box and Blocks Tests (BBT): (**a**) components: wooden box and colored cubes; (**b**) mechanics: user performing the test.

The Box and Blocks Test (BBT) is an outcome measure widely used in neurological rehabilitation to quantify the upper limb motor function, especially the level of gross manual dexterity and coordination [11]. Figure 1 depicts the components of the system and the movements of the patient when performing the test. The physical elements of the BBT are a wooden box with two 290 mm wide square compartments and 150 wooden 1-in cubes. Additionally, a 100 mm high partition is located between the two compartments to separate them (see Figure 1a). To administer the BBT, it is necessary to place the patient in front of the box, the two wooden compartments must remain in the mid-line of the patient, and the patient must move the cubes from one side to the other, finally, it is important that the therapist gives them the correct instructions before starting the test.

The mechanics of the test encourage the patient to move as many cubes as possible, one at a time, from one compartment to the other in one minute (see Figure 1b). Once the time is completed, the therapist manually must count the number of transported cubes in order to calculate the score. The higher the number of cubes, the higher the level of manual dexterity.

The evaluation process is conducted in three phases: (a) a training trial of fifteen seconds to help the patient to become familiar with the test, (b) the assessment of the dominant (less affected) hand, and (c) the assessment of the non-dominant (more affected) hand.

Although obtaining the final score (number of cubes) seems simple, the therapist must also pay attention to the patient as the test is being performed to identify any errors the patient makes. In this sense, care must be taken that the patient does not put the whole hand on the other side when leaving the cube, or that he/she does not take more than one cube in the hand, for example. If the patient made any of these errors, the cube would not be counted in the final score, so his score would be reduced.

On account of the above, it is possible to identify some drawbacks on functional assessment based on the traditional BBT. These drawbacks primarily come from the manual development of the BBT. Here, one relevant issue is the observation-based rating of attempts to validate the displacement of cubes, according to the BBT rules. This validation tries to be reliable and objective; however, the test development includes some degree of variability as it is based on an observation of the task, so several of these limitations could be reduced through the automation of the test.

### Related Work

The state-of-the-art approaches in the automation of the BBT presents several approaches to obtain its score automatically. One strategy is the use of a depth-sensing camera to capture frame-by-frame the cubes displacement [26,27]. These frames are processed using computer vision techniques to estimate the number of transferred cubes. This strategy not only provides the therapist with an objective cube counting method but also with additional information about the user’s performance. The success rate of this type of system is high, even in clinical trials with patients with PD [28]; however, a remaining issue is to provide a method for a more autonomous administration of the test.

Another strategy that also employs depth-sensing cameras is the use of gaming technology to build a virtual environment, which can be used to measure the user’s interaction. As an example, in the study conducted by Cho et al. [22], a virtual version of the BBT based on the Microsoft Kinect V1 sensor was created. This system is able to detect the movements of hand, fingers, and a grasping gesture. Clinical trials with this system showed good results with stroke survivors; however, it has some issues to solve. Firstly, this system was designed to run in a flat-screen monitor setup, which can limit the depth perception for users; furthermore, BBT development includes fine manual capabilities (pinching) and, hence, it requires proper hand gesture recognition. The Kinect sensor has limitations to detect fingers gestures. Ultimately, the automatic test administration was not studied. Problems in hand gesture recognition were improved in the virtual version of the BBT presented in the study conducted by Gieser et al. [23] by means of using the leap motion controller (LMC) for hand motion tracking. The use of LMC highly improves the detection of precise hand movements and showed a good performance with healthy users. Nevertheless, this system was still running in a desktop mode (with a flat-screen), and the automatic test administration was not considered.

One concern about the use of VR systems could be the associated cost of devices (game-compatible PC, headset, and sensors), which will be higher than the price of the BBT box; however, the cost of VR equipment has reduced significantly since its introduction to the marketplace, and is now quite affordable. Contrary to the physical BBT, VR equipment can be used in diverse applications of rehabilitation, as it is a powerful suite for training or evaluating various functional impairments under the supervision of clinicians [29]. This multipurpose capability of VR technology is the main motivation for developing healthcare-related systems using VR.

As has been shown, previous virtual versions of the BBT have been piloted with healthy subjects [23] and stroke survivors [22]. In both studies, the scores obtained with the virtualized versions were lower than the ones obtained from the real BBT; however, the correlation analysis between the physical and virtual systems was high in both studies. On this basis, in a preliminary study [25], we tested our proposed fully-immersive virtual version of the BBT obtaining similar results with a PD cohort. In this paper, we extended the size of the sample to validate the preliminary conclusions and conduct further analysis of data from trials. The following section describes in deep the features and advantages of our proposed VR-BBT.

## 3. The VR-Based Box and Blocks Test

The VR-based Box and Blocks Test (VR-BBT) is a fully immersive virtual version of the BBT that combines the classical BBT mechanics with a play-centric approach to achieve a fully automated system for evaluating hand motor function [24]. The principal characteristics of the VR-BBT include:

- robust tracking of fine hand movements by using the LMC sensor;
- gameplay oriented to the automatic test administration;
- an automatic and objective score generation according to the traditional test mechanics;
- better depth perception using a 3D virtual workspace.

**Figure 2:**
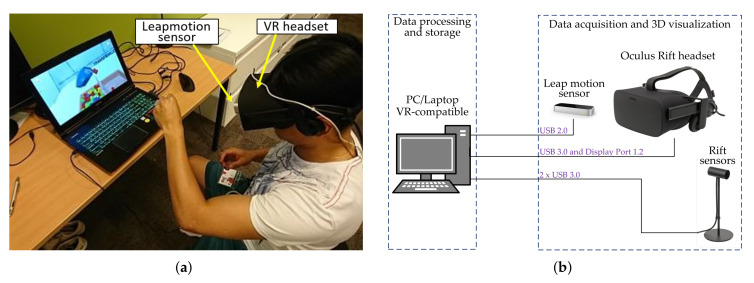
The virtual reality (VR)-based Box and Blocks Test (BBT): (**a**) assessment setup and components; (**b**) hardware connection of devices.

Figure 2a illustrates the assessment setup of the VR-BBT, including its components. For the UE evaluation, the user must be seated to safely wear the Oculus Rift headset. Since the user stays in a seated position, the risk of falling due to possible dizziness is minimized. The LMC sensor is placed in the Oculus Rift headset for working in the head-mounted configuration. A gaming PC is required to run the virtual environment in the VR headset. Figure 2b presents the hardware connection of devices. The virtual environment was implemented using the Unity 3D software, and the Interaction Engine module was employed to improve the gesture recognition capabilities of the LMC.

### 3.1. System Environments

**Figure 3:**
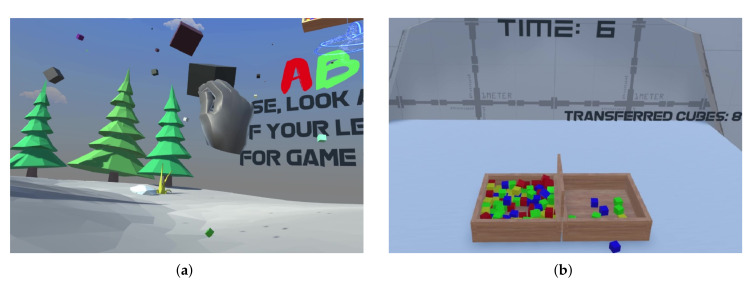
Virtual scenarios of the VR-BBT: (**a**) welcome scenario; (**b**) assessment scenario.

The VR-BBT system integrates two virtual scenarios: the first one focused on helping the user to become familiar with the virtual reality, and the second one focused on the autonomous assessment of manual dexterity. Figure 3 presents the two scenarios offered by the VR-BBT (https://www.youtube.com/watch?v=l_h1xz_cBoI) system: (1) welcome scenario, and (b) assessment scenario.

The welcome scenario (see Figure 3a) aims to help the user to become familiar with the immersion in 3D-VR and, particularly, in the grasping of virtual cubes. For that purpose, this scenario is represented by a forest where several virtual cubes are randomly moving around the user’s location. The user is free to catch and handle any cube. Since the purpose of this scenario is to motivate the user, the size of the virtual cubes is double that of the BBT’s ones in order to facilitate the initial contact. The physics of the virtual cubes were designed to simulate a reduced gravity, providing a simple environment for the natural interaction with the hands. In this way, the user can play with the cubes by making them spin, throwing them, or making them explode when crashing into each other. This scenario has no rules nor score.

The assessment scenario (see Figure 3b) is intended to perform the UE assessment in the same way as the conventional BBT. This way, the assessment process involves the grasping of virtual cubes one-by-one and moving them to the contrary compartment. Consequently, we built virtual versions of the box and cubes, keeping the dimensions of the physical ones. The proposed assessment scenario is formed by elements such as the BBT box with two compartments, interactive components (buttons), information panels to display the time, score, and instructions, and a clear–grey table to imitate a desk and sustain the BBT box. Finally, this scenario provides the total amount of cubes transferred and that have accomplished the test’s rules, in order to (a) facilitate the test administration and (b) objectively measure (rate) the attempts.

### 3.2. Test Administration Process

**Figure 4:**
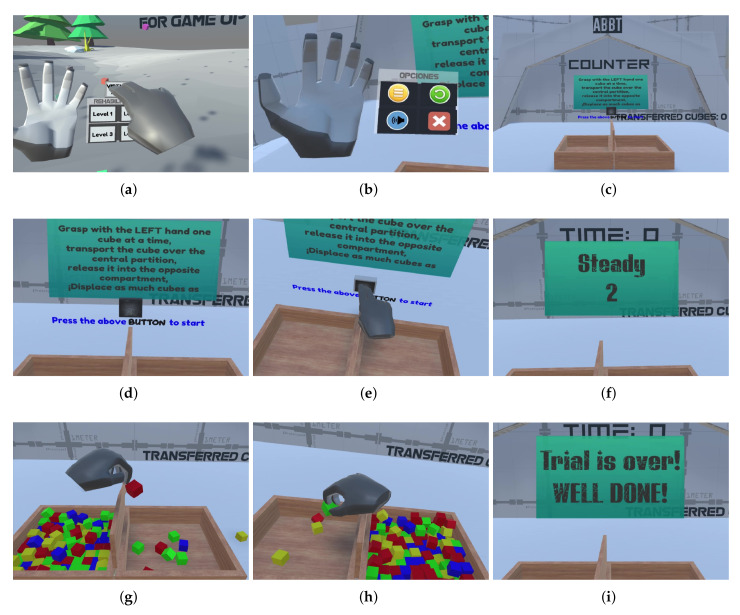
Process for automatic test administration: (**a**) launching assessment; (**b**) options’ menu; (**c**) assessment scenario; (**d**) instructions given; (**e**) starting evaluation; (**f**) intermediate panels; (**g**) dominant hand evaluation; (**h**) non-dominant hand evaluation; (**i**) farewell panel.

The automatic test administration in the VR-BBT was addressed through a pre-programmed gameplay and interactive navigation menus (see Figure 4) to guide the user in all evaluation stages. The flow (steps) when developing the VR-BBT is described as follows.

From the welcome scenario, the user must launch the assessment scenario using the navigation panel attached to the virtual right hand. The navigation panel is available when the user looks at the palm opening its hand (Figure 4a). Then, the user can choose the corresponding option using the left hand. Note that the navigation panel is also available in the assessment scenario (Figure 4b), allowing users to go back to the welcome scenario, repeat an attempt, turn up the volume, or exit the VR-BBT.

Once the user has entered the assessment level, the virtual BBT box appears in front of the patient (Figure 4c). Note that the box has two empty compartments. Then, the test instructions are provided to the user through the text in the instructions panel (Figure 4d) and audio messages. Then, the user must push the square black button (Figure 4e) in order to confirm the understanding of instructions and be ready to start the first assessment stage. After pushing the button, a regressive counter (Ready, Steady, Go) is activated and displayed on the front panel (Figure 4f).

Starting with the more affected side, 150 virtual cubes will appear at the corresponding compartment (left side in Figure 4g). Note that the more affected side is indicated at the beginning in the user’s information window where the personal data is collected.

While the test timer is running, the user must grasp any cube, move it to the other compartment, and drop it. The grasping action of virtual cubes was designed for natural interaction. That means the user can use a pinching gesture with the thumb and index fingers, grasping with three fingers (thumb, index and middle fingers), or a fist gesture. In all the above cases, the mechanics are similar to the real BBT since the simulated physics of the virtual objects do not allow the cube to pass through the barrier. Thus, the virtual environment forces the users to overcome the central barrier with their hand to score.

When this stage is completed (after 60 s), the final score is displayed in the result panel. This above sequence is automatically repeated for the case of the less affected hand (right side in Figure 4h). At the end of both assessment stages, a farewell message (Figure 4i) is displayed and the welcome scenario is launched again.

### 3.3. Score Validation

As the number of virtual cubes transferred is the principal outcome, validation of the attempts is a fundamental point. An attempt is valid when accomplishing the BBT rules; for instance: the user’s hand must overcome the central partition, the cube must fall inside the opposite compartment, and one cube at a time. The accomplishment of the above conditions can be interpreted from measuring the interactions into the virtual environment. For that purpose, several collision detectors, denoted as virtual colliders, were carefully placed to identify events such as the grasping of more than one cube at a time or the hand position when releasing the cube. This way, the VR-BBT detects objectively whether the rules were accomplished to mark an attempt as valid. Cubes falling out the compartment, cubes falling inside without the user’s hand overcoming the central partition (throwing the cube), or transporting more than one cube at a time are registered as invalid attempts. The scores are automatically stored in a CSV file for each user. Additionally, hand trajectories are also saved in the same file for further analysis.

## 4. Pilot Study Description

The pilot study was carried out at the Association of Patients with Parkinson’s disease APARKAM in Alcorcón, Madrid (Spain). The sample recruited was twenty patients with PD. This research was approved by a local ethics committee, and informed consent was obtained from all individuals included in this research.

In this trial, each participant was asked to perform both the physical BBT (once) and the VR-BBT (twice). The order of the tests performed was: (1) physical BBT, (2) VR-BBT (attempt 1), and (3) VR-BBT (attempt 2). The virtual system was tested twice to analyze the test–retest reliability of the VR-BBT. A rest period was given to the participants between attempts. The order of participants to perform the trial was randomly defined according to the time-schedule that best fit each participant.

The administration of the physical BBT was carried out according to the instructions of the test [11]. The participants had to move the cubes to the other side of the box for 60 s and the evaluator counted at the end of the time the cubes that had been passed. Note that the physical BBT was performed once, and a rest period of five minutes was given before testing the VR-BBT.

Regarding the administration of the VR-BBT, each participant went through a fitting process to comfortably wear the Oculus Rift headset and tune the lens alignment for best optical focus before testing. This fitting process took roughly five minutes on average for each user. Once the headset was properly fitted, participants were free to interact in the welcome scenario for five minutes. Afterwards, participants were instructed verbally to pick up and move as many virtual cubes as possible in the assessment scenario. Participants were reminded to overcome the virtual central barrier with their hand. Hence, instructions provided were similar to the ones recommended for the physical BBT. The rest period between the attempts with the less affected and more affected hand was one minute. Two attempts with the VR-BBT were conducted in order to estimate the test–retest reliability. A ten minute rest period was given between the two attempts.

### 4.1. Participants

The total sample was composed of twenty individuals with PD, seventeen men and three women. Table 1 shows the socio-demographic data of the sample. The mean age of the patients was 74.38 ± 0.94 years. In fourteen patients, the most affected side was the left side, in the remaining six, the right side was the more affected.

The inclusion criteria, following our previous research [25], were as follows: (1) patients with PD fulfilling the modified diagnostic criteria of the Brain Bank of the United Kingdom, (2) PD in stages II, III, and IV of the Hoehn and Yahr scale, (3) scoring over 60% in Schwab and England functionality scale, (4) stable or slightly fluctuating motor response to pharmacological treatment, and (5) individuals not receiving specific UE rehabilitation treatment at the time of the study.

The exclusion criteria, according to our previous research [25], were as follows: (1) diagnosis of diseases other than PD or serious injuries affecting the UE, (2) scoring lower/equal than 24 points in the Mini-mental Test, (3) refusal to participate in the study, (4) PD in stages I or V of the Hoehn and Yahr scale, and (5) visual impairment not correctable by glasses.

### 4.2. Statistical Analysis

Statistical analysis was performed using SPSS software for Windows (SPSS Inc., Chicago, IL, USA; version 25.0). Pearson’s correlation coefficient was used to analyze the relationship between BBT (number of cubes) and the first evaluation (attempt 1) carried out by the VR-BBT (number of virtual cubes). Pearson’s correlation coefficient was used to analyze the relationship between the Hoehn and Yahr scale and the VR-BBT attempt 1. The correlation for the coefficients from 0.00 to 0.49 was interpreted as poor, those from 0.50 to 0.79 as moderate and those from 0.8 or more as excellent [30].

The intraclass correlation coefficient (ICC) was used to analyze the test–retest reliability of the VR-BBT and its 95% confidence interval, using a mixed effects model, absolute agreement [31,32]. The number of cubes was analyzed in the two evaluations carried out by the VR-BBT. Values greater/equal than 0.6 were considered excellent, ICC values between 0.31 and 0.59 were considered suitable, and values lower/equal than 0.30 were considered unreliable. Statistical significance of *p* <0.05 was established for all the tests.

### 4.3. Satisfaction Assessment

Clinicians’ usability and acceptability perceived with the VR-BBT system were evaluated by a satisfaction questionnaire [28]. Three clinicians, experts on Parkinson’s disease neurorehabilitation answered four questions and rated them on a Likert-type scale from 1 to 5 (strongly disagree—strongly agree). The arithmetic mean across all items provides the total satisfaction score.

Similarly, the patients’ satisfaction and acceptability perceived when using the VR-BBT system were evaluated by a satisfaction questionnaire designed by the research team. In this case, patients answered five questions and rated them on the same Likert-type scale from 1 to 5 (strongly disagree—strongly agree). The arithmetic mean across all items provides the total scores of satisfaction and acceptability.

## 5. Results

The number of cubes displaced by each participant for the case of the physical BBT and the VR-BBT are summarized in Table 2, according to the more and less affected hand. Table 2 also presents the average of displaced cubes in the physical BBT and virtual VR-BBT systems. It can be seen that participants scored higher using the physical BBT. This difference was quantified through a systematic error (SysErr) given by the coefficient between the average of transferred cubes from the BBT and the average of transferred cubes from the VR-BBT, according to the more and less affected hand.

Table 3 presents the ratio percentages given by: (number of cubes moved by the more affected side) ÷ (number of cubes moved by the less affected side) × 100. Numerical values obtained for each participant are summarized in Table 3a, while Table 3b presents a scatter plot of such values for better visualization.

**Figure 5:**
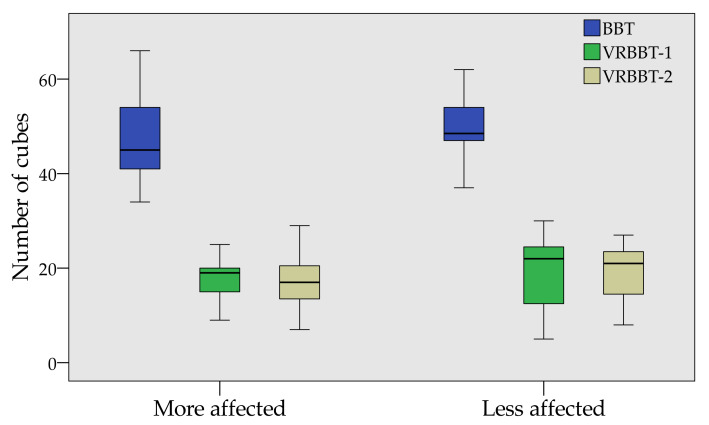
Box-plot of the BBT and VR-BBT scores.

Figure 5 summarizes, in a box-plot, the scores obtained by each participant according to both the less and more affected hand. The blue box represents the scores from the physical BBT, the green box represents the results from the first attempt of the VR-BBT, and the ochre box represents the scores from the second attempt of the VR-BBT.

It can be noted that all of the individuals moved more cubes in the physical BBT than the virtual system; however, a similar number of virtual cubes were transported between the first and second attempts of the VR-BBT.

**Figure 6:**
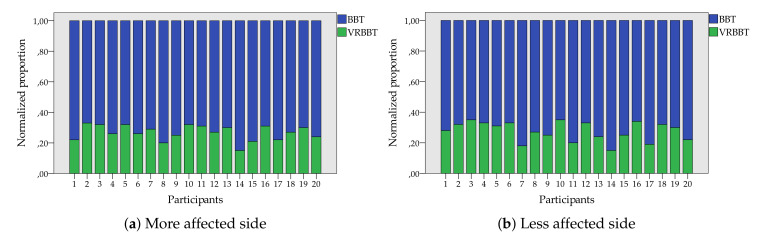
Normalized proportion of transferred cubes with the BBT and VR-BBT systems.

Figure 6 illustrates the normalized proportion in cube displacement for the case of the physical and virtual systems, according to the more and less affected hand. It can be seen that all of the participants moved more physical cubes (blue bar) than virtual ones (green bar); however, the proportion between both systems is roughly stable around 25%.

### 5.1. Reliability Analysis

**Figure 7:**
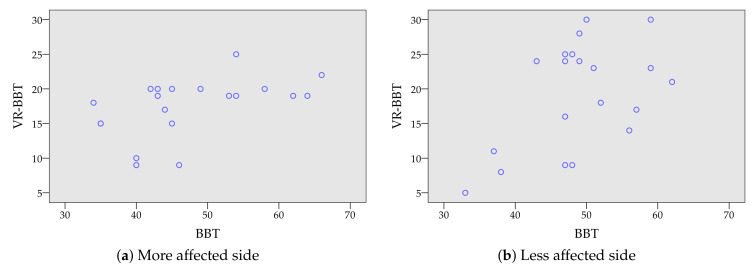
Correlation plot between virtual scoring (VR-BBT) and manual counting (BBT).

Figure 7 presents in a scatter plot the correlation level between the number of cubes transferred with the physical BBT and the ones transferred in the first attempt of the VR-BBT. Results are presented according to the more and less affected side in Figure 7a,b, respectively. It can be appreciated a high dispersion in the plotted sample. This variance could come from the difference between the physical and virtual grasping of cubes.

Pearson’s correlation coefficients between the scores from the BBT and the VR-BBT systems are presented in Table 4. The coefficients showed a statistical significance for both sides (*p* =0.025 for the more affected side and *p* =0.022 for the less affected side), with a moderate correlation for the more affected side (*r*=0.499) and the less affected side (*r*=0.510).

Pearson’s correlation coefficients between the Hoehn and Yahr scale and the VR-BBT system are described in Table 5. Statistical significance was found in the more affected side (*p* = 0.001) and in the less affected side (*p* = 0.038) with a moderate correlation (*r* = 0.496 and *r* = 0.696, respectively).

The results of test–retest reliability of the VR-BBT showed statistical significance between the two attempts for the most affected side (*p* <0.001) and for the less affected side (*p* <0.001) with an excellent correlation for both cases (ICC = 0.876; ICC = 0.873, respectively). These results are presented in Table 6.

### 5.2. Usability Assessment

The perceived usability and acceptability of the VR-BBT proposed presented high to excellent results. The clinicians’ answers are summarized in Table 7. Two clinicians evaluated “strongly agreed” and one “agreed” with the satisfaction with the VR-BBT. Two clinicians reported that the VR-BBT was useful in order to assess unilateral gross manual dexterity (“strong agreement”), but the other one reported “agreed” in this regard. Regarding the degree of recommendation of the VR-BBT, all clinicians reported to be in “strong agreement”. All raters showed a strong agreement with the advantages of the VR-BBT compared to the BBT. Considering the scale (strongly disagree = 1 point to strongly agree = 5 points), the mean score in the raters’ satisfaction scale was: 4.83 points.

The results for the satisfaction and acceptability questionnaires answered by the patients with PD are summarized in Table 8. This table shows the number of participants that marked a specific answer. Patients’ satisfaction and acceptability of the VR-BBT rated by the patients were as follows: mean score for question 1 was 4.70 points; 4.70 points for question 2; 4.85 points for question 3; 4.40 points for question 4; and 4.75 points for question 5. The overall mean score for patients’ satisfaction and acceptability of the VR-BBT was 4.68 points. Based on the overall mean score obtained in questionnaires, the acceptance of the VR-BBT by the patients with PD was considered very high.

## 6. Discussion

This study focused on the validation of a fully-immersive virtual version of the BBT as a feasible method for evaluating the UE motor function in patients with PD. Our VR-BBT system was piloted with a convenient sample of twenty participants in order to consolidate the promising conclusions obtained in a preliminary study [25]. Thus, the present study showed three relevant findings: (1) the gap in the number of cubes transported in the physical and virtual systems seem to tend to a constant (systematic error), (2) the correlation between the outcome obtained with the physical and virtual systems is statistically significant, and (3) the test–retest analysis shows an excellent correlation, and statistically significant, between the attempts with the virtual system. The results of this study agree with the preliminary ones, are in line with other research [22,23], and provide relevant information about the performance of the VR-BBT system regarding the conventional assessment.

Firstly, all the participants scored higher with the physical BBT. In particular, the number of transported cubes in the physical system was about 2.5 times and 2.7 times higher than in the VR-BBT, for the less and more affected hand, respectively. This fact interestingly agree with previous studies in other populations as healthy subjects [23] or stroke survivors [22]. In those studies, the difference in the number of cubes transported was 2.8 times for the case of healthy students and 3 times for the case of stroke survivors. On this basis, and considering our obtained results, it seems that the assumption that a systematic error exist is true and, therefore, the gap between the physical and virtual systems could tend to a constant.

In this sense, the relationship between the physical and virtual systems can be enhanced (make closer) by using fully-immersive VR thanks to the better perception of depth. Note that the systematic error obtained with our virtual system is lower than related work where fully-immersive VR was not used. Hence, the results point to that the 3D depth perception allowed for displacing more virtual cubes in our VR-BBT. The use of the LMC could also contribute to this fact. Despite the good results, more investigation is required to understand the way that the LMC affects the absence of tangible feedback in cube transferring, and the exploration of novel methods to balance the loss of tactile feedback.

Secondly, the statistical analysis of scores from the physical BBT and the VR-BBT showed a statistical significance for both the more and less affected sides (p=0.025 and p=0.022, respectively), and even for the whole sample (p=0.002); however, the correlation level is moderate in all cases. This correlation level agrees with the findings of a similar study with stroke survivors [22] that suggest the difference between physical and virtual systems lays on the nature of virtual reality. In the case of the physical BBT, the performance in cube displacement is affected by grip surfaces [33]. Thus, both virtual and physical systems may be fundamentally different; however, the high level of significance obtained in our statistical analysis proves that despite the differences, the virtual system could maintain the clinical meaningfulness of the conventional test.

We have studied the correlation level of the VR-BBT regarding a specific outcome measure used in PD by using the Hoehn and Yahr scale. Our results show a moderate correlation between the Hoehn and Yahr scale and VR-BBT (attempt 1) for the more and less affected sides (*r* = 0.496 and *r* = 0.696, respectively). Previously, Hwang and Song [34] investigated the relationship between manual dexterity and the Unified Parkinson’s Disease Rating Scale (UPDRS) motor exam, using the conventional BBT in people with PD. Their results showed that the UPDRS-motor exam score presented a negative correlation with the BBT for both sides. Similarly, our results suggest that the VR-BBT is correlated with the severity of the disease measured by the Hoehn and Yahr scale, for both sides. This fact indicates that the VR-BBT could be a good clinical measure to quantify upper extremity dexterity in patients with PD in mild to moderate stages of the disease.

Additionally, by analyzing the ratio percentages in cube displacement, a relationship was not found between the ratio percentages in the VR-BBT with those in the BBT. This fact is contradictory with the strong correlation found in the study conducted by Cho et al. [22] that used a smaller sample (nine participants). The difference may lay in the sample size and population characteristics. Thus, more research is needed to study the effect of patient’s demographics.

Finally, the test–retest analysis showed an excellent association level between the two attempts of the VR-BBT. The correlation level is significant for the case of both the more and less affected sides. This fact indicates that saving the differences between virtual and physical systems the VR-BBT can be a reliable method to assess the UE motor function in people with PD in a mild to moderate stage of the disease. Therefore, the findings of this study suggest that our VR-BBT can consistently measure the evolution of hand motor function, being a method more friendly and encouraging than conventional one.

Related to clinicians’ satisfaction, all raters were highly satisfied with the VR-BBT as an assessment tool for upper limb motor function in patients with PD. Some concerns were identified related to the first uses in VR scenarios as an evaluating method in these type of patients or patients with glasses; however, most patients did not find difficulties in performing the test in VR environment as compared to the traditional BBT, as instructions and upper limb movements were easy to understand, and results were comparable to the BBT. All raters would recommend the use of the VR-BBT to other clinicians in the PD context. The main advantages provided by the VR-BBT are its portability, ease of use, customizable VR assessment scenarios, and non-invasive nature. On account of the above, the possibility of using VR technology to develop automatic assessment systems of UE motor function in general, and manual dexterity in particular, seems feasible. This assumption also is supported by previous work conducted by our research team on the validation of the automated version of the BBT using a Microsoft Kinect sensor [28].

Regarding acceptance, previous studies have described the use of this type of research in patients with PD by combining the analysis of the effects of experimental interventions or assessments with qualitative investigations about the impact of patient acceptance or rejection of the regarding the application of these technologies [35,36,37,38]. In the present study, patients’ perceptions were assessed to obtain information about satisfaction and acceptability with the VR-BBT rated by the patients. The mean score showed very high satisfaction and acceptability perceived by the patients. The items rated with higher scores were those related to satisfaction with the services received and usefulness for rehabilitation. The item with a lower score was related to the difficulty of the adaptation and management of the technology. This fact is consistent with our previous work on experience after using novel immersive VR technology in patients with PD [39]. So, future studies should take into account these results.

Our study had several limitations. First, the sample size was limited and there was no control group. We only included patients with PD in mild to moderate stages of the disease, so no extrapolations to other neurological conditions and stages of the disease can be done. All patients were in a phase of their medication cycle. Finally, we did not use the UPDRS to explore PD patients’ severity, so future studies should be conducted to explore the correlation between our VR-BBT system and UPDRS III (motor section). to our knowledge, this is the first paper to examine the feasibility and psychometric properties of immersive applications of the BBT in patients with PD in mild to moderate stages of the disease.

## 7. Conclusions

This paper described the validation of a fully immersive VR-based system based on the Box and Blocks Test to reliably measure the manual dexterity in a convenient sample of participants with PD. This study involves a technical assessment where the scores obtained with a VR-BBT were compared with the physical BBT scores. The results showed a statistically significant association between the number of cubes obtained in both systems, and a correlation between the Hoehn and Yahr scale and the VR-BBT. Furthermore, there is a systematic gap between the scores from the physical and virtual systems around 2.8 times; however, this difference can be reduced by using a fully-immersive system as in the present study. Additionally, the test–retest analysis for the scores obtained from the VR-BBT showed an excellent correlation. These results demonstrate that the VR-BBT might be valid and reliable for the assessment of manual dexterity in PD. More importantly, this evaluation suggests that this VR-BBT system has the potential to be used in clinical settings as it can consistently measure both spatial and time-based parameters required for the fulfillment of a validated clinical test, with high usability, acceptability, and satisfaction rated by clinicians and patients with PD.

## Figures and Tables

**Table 1:** Socio-demographic and descriptive data of participants.

Variable	Data
Age ${}^{†}$	74.35 (±0.94)
Sex (male/female)	17/3
More affected side (right/left)	6/14
Hoehn & Yahr	II (8) /III (12)
Schwab and England score (%) ${}^{†}$	71.66 (± 4.01)

${}^{†}$ Mean (± SD).

**Table 2:** Total scores obtained with the physical and virtual systems; the systematic error (SysErr): (average of transferred cubes with the BBT) ÷ (average of transferred cubes with the VR-BBT).

Participant	More Affected Side				Less Affected Side			
	BBT	VRBBT			BBT	VRBBT		
		Attempt 1	Attempt 2	Mean		Attempt 1	Attempt 2	Mean
1	66	22	15	18.5	59	23	23	23.0
2	54	25	29	27.0	59	30	26	28.0
3	45	20	23	21.5	49	28	24	26.0
4	58	20	21	20.5	49	24	25	24.5
5	35	15	18	16.5	37	11	22	16.5
6	54	19	19	19.0	51	23	27	25.0
7	35	15	14	14.5	47	9	12	10.5
8	40	9	11	10.0	52	18	21	19.5
9	62	19	22	20.5	57	17	21	19.0
10	43	20	20	20.0	50	30	24	27.0
11	34	18	13	15.5	38	8	11	9.5
12	53	19	21	20.0	48	25	22	23.5
13	43	19	17	18.0	47	16	13	14.5
14	46	9	7	8.0	48	9	8	8.5
15	64	19	15	17.0	62	21	21	21.0
16	42	20	17	18.5	43	24	21	22.5
17	40	10	12	11.0	33	5	10	7.5
18	49	20	17	18.5	47	24	21	22.5
19	44	17	20	18.5	47	25	16	20.5
20	45	15	13	14.0	56	14	18	16.0
**Avg.**	48			17.4	49			19.3
**SD**	9.6			4.3	7.5			6.4
**SysErr**				**2.76**				**2.54**

**Table 3:** Ratio percentage for the physical and virtual systems; Ratio percentage: (number of cubes moved by the more affected side ÷ number of cubes moved by the less affected side) × 100.

**(a) Ratio Percentage**			**(b) Scatter Plot**
**Participant**	**Ratio (%)**		
	**BBT**	**VRBBT**	
1	111.86	80.43	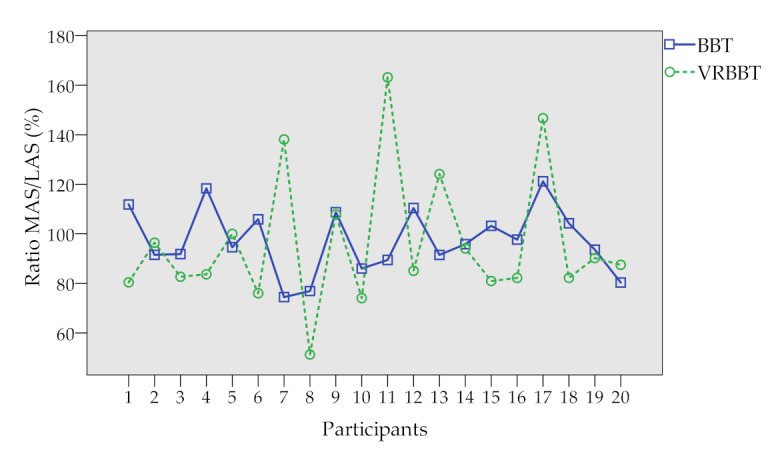
2	91.53	96.43	
3	91.84	82.69	
4	118.37	83.67	
5	94.59	100.00	
6	105.88	76.00	
7	74.47	138.10	
8	76.92	51.28	
9	108.77	107.89	
10	86.00	74.07	
11	89.47	163.16	
12	110.42	85.11	
13	91.49	124.14	
14	95.83	94.12	
15	103.23	80.95	
16	97.67	82.22	
17	121.21	146.67	
18	104.26	82.22	
19	93.62	90.24	
20	80.36	87.50	

**Table 4:** Pearson’s correlation coefficients between physical (BBT) and virtual (VR-BBT) systems.

	BBT—VRBBT *n* = 20	
**Measure**	***r***	***p***
More affected side	0.499	0.025 *
Less affected side	0.510	0.022 *

* Significant at the 0.05 level (2-tailed).

**Table 5:** Pearson’s correlation coefficients between the Hoehn and Yahr scale and VR-BBT systems.

	Hoehn & Yahr Scale—VRBBT	
	*n* = 20	
**Measure**	***r***	***p***
More affected side	0.696	0.001 *
Less affected side	0.466	0.038 *

* Significant at the 0.05 level (2-tailed).

**Table 6:** Test–retest reliability of the VR-BBT.

	ICC	IC95%	*p*
More affected side	0.876	0.686–0.951	<0.001 *
Less affected side	0.873	0.675–0.950	<0.001 *

* Significant at the 0.001 level.

**Table 7:** Results of satisfaction questionnaires for clinicians.

Clinicians	Items ${}^{†}$	Strongly Disagree	Disagree	Neither Agreement Nor Disagreement	Agree	Strongly Agree
1	(1)					x
	(2)					x
	(3)					x
	(4)					x
2	(1)					x
	(2)					x
	(3)					x
	(4)					x
3	(1)				x	
	(2)				x	
	(3)					x
	(4)					x

${}^{†}$ Items: (1) Are you satisfied with the VR-BBT? (2) Has the VR-BBT been useful in order to assess unilateral gross manual dexterity? (3) Would you recommend the VR-BBT to other clinicians? (4) Do you think that the VR-BBT has advantages compared to the BBT?

**Table 8:** Results of satisfaction questionnaires for participants.

	Items ${}^{†}$	Strongly Disagree	Disagree	Neither Agreement Nor Disagreement	Agree	Strongly Agree
(1)	Would you recommend this assessment to other patients?			2	2	16
(2)	Would you repeat this assessment if your clinician recommends it?			1	4	15
(3)	Overall, I am satisfied with the services received				3	17
(4)	The adaptation and management of technology has been easy		2	3		15
(5)	I consider this type of assessment useful for rehabilitation			2	1	17

${}^{†}$ Scores are given as the number of participants who marked an item.

## Abbreviations

The following abbreviations are used in this manuscript:

BBT	Box and Blocks Test
LMC	Leap motion controller
PD	Parkinson’s disease
UE	Upper extremity
VR-BBT	Virtual Reality-based Box and Blocks Test
VR	Virtual Reality
